# The D-amino acid peptide D3 reduces amyloid fibril boosted HIV-1 infectivity

**DOI:** 10.1186/1742-6405-11-1

**Published:** 2014-01-14

**Authors:** Marek Widera, Antonia Nicole Klein, Yeliz Cinar, Susanne Aileen Funke, Dieter Willbold, Heiner Schaal

**Affiliations:** 1 Institut für Virologie, Heinrich-Heine-Universität, D-40225 Düsseldorf, Germany; 2 Forschungszentrum Jülich, ICS-6, Jülich, 52425, Germany; 3 Institut für Physikalische Biologie, Heinrich-Heine-Universität, Düsseldorf, 40225, Germany; 4 Bioanalytik, Fakultät für Angewandte Naturwissenschaften, Hochschule für Angewandte Wissenschaften Coburg, 96450 Coburg, Germany; 5 Biologisch und Medizinisches Forschungszentrum (BMFZ), Heinrich-Heine-Universität Düsseldorf, Düsseldorf, Germany

**Keywords:** HIV-1 infection, SEVI, D3, Amyloid-beta, Alzheimer’s disease, D-enantiomeric peptide, Drugs, Monomers, Oligomers

## Abstract

**Background:**

Amyloid fibrils such as Semen-Derived Enhancer of Viral Infection (SEVI) or amyloid-β-peptide (Aβ) enhance HIV-1 attachment and entry. Inhibitors destroying or converting those fibrils into non-amyloidogenic aggregates effectively reduce viral infectivity. Thus, they seem to be suitable as therapeutic drugs expanding the current HIV-intervening repertoire of antiretroviral compounds.

**Findings:**

In this study, we demonstrate that the small D-amino acid peptide D3, which was investigated for therapeutic studies on Alzheimer’s disease (AD), significantly reduces both SEVI and Aβ fibril boosted infectivity of HIV-1.

**Conclusions:**

Since amyloids could play an important role in the progression of AIDS dementia complex (ADC), the treatment of HIV-1 infected individuals with D3, that inhibits Aβ fibril formation and converts preformed Aβ fibrils into non-amyloidogenic and non-fibrillar aggregates, may reduce the vulnerability of the central nervous system of HIV patients for HIV associated neurological disorders.

## Findings

Amyloid fibrils exhibiting a cationic surface [1], for example those of the Alzheimer’s disease (AD) related amyloid-β peptide (Aβ) and the Semen derived Enhancer of Viral Infection (SEVI), promote HIV infection by facilitating viral attachment through neutralization of the electrostatic repulsion between the negatively charged surface of virions and target cells [2-4]. Experimental approaches to reduce SEVI-mediated enhancement of HIV-1 infection by amyloid binding agents have already been described [5-9]. However, except for epigallocatechin-3-gallate, the major active constituent of green tea, most of these compounds were shown to bind, but not to eliminate amyloids. Recently, it was demonstrated that the small D-amino acid peptide D3 converts Aβ oligomers and fibrils into non-amyloidogenic, non-fibrillar and non-toxic aggregates and reduces the cognitive deficits of the central nervous system in transgenic AD model mice [10]. Because many amyloid fibrils, despite their composition of different peptides or proteins, show significant structural similarities like a typical cross-beta sheet quaternary structure, we intended to analyze the inhibitory capacity of D3 to reduce other amyloid caused pathologic effects.

**Figure 1:**
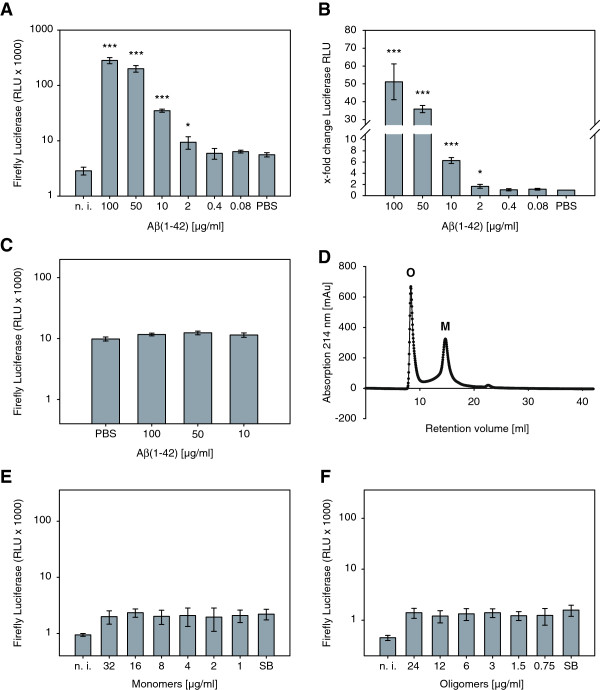
**Aβ(1–42) fibrils but not mono- and oligomers enhance HIV-1 infection of TZM-bl cells. (A)** Equal amounts (500 TCID_50_ as determined with TZM-bl cells using supernatant of transfected HEK 293T cells) of the dual-tropic HIV-1 lab strain NL4-3 PI 952 [11] were pre-incubated for 5 min at RT with Aβ(1–42) fibrils. Subsequently, the pretreated viruses were used to infect TZM-bl reporter cells and infection-induced luciferase activity was assayed 48 h post infection. (*** p < 0.001, * p < 0.05 referred to PBS treated and infected cells). **(B)** X-fold change of luciferase enhancement was quantified relative to cells infected in the absence of Aβ(1–42) fibrils (PBS). **(C)** Luciferase RLUs of non-infected cells, which were treated with the indicated concentrations of Aβ(1–42). **(D)** Chromatogram of a size exclusion chromatography (SEC) showing the absorption profile of Aβ(1–42) monomers (M) and oligomers (O), which were used in the following analysis. **(E and F)** The same experiments as in **(A)** but viruses were pre-incubated with Aβ(1–42) mono- and oligomers obtained by SEC shown in **(D)**. milli-absorbance-units (mAu); non-infected (n.i.); relative light units (RLU); size exclusion chromatography buffer (SB).

**Figure 2:**
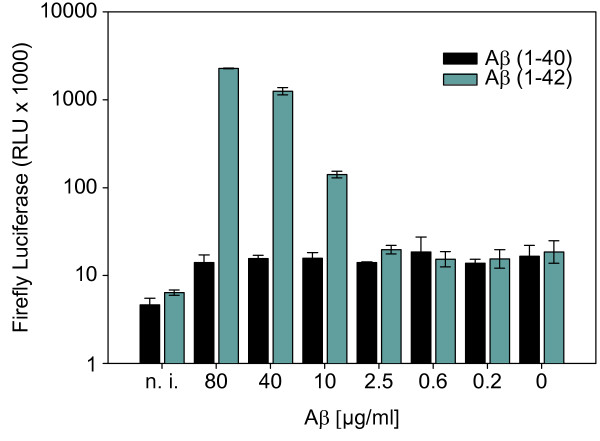
**Aβ(1–42) but not Aβ(1–40) fibrils enhance HIV-1 infection of TZM-bl cells.** Equal amounts of the dual-tropic HIV-1 lab strain NL4-3 PI 952 [11] were pre-incubated for 5 min at RT with the indicated concentrations of Aβ(1–42) or Aβ(1–40) fibrils, which were incubated for four and six days, respectively of oligomerization. Subsequently, the pretreated viruses were used to infect TZM-bl reporter cells and infection-induced luciferase activity was assayed 48 h post infection.

In order to utilize amyloidogenic inhibitors to reduce fibril boosted viral infectivity, we firstly wanted to unravel whether fibrils or even monomers or oligomers of Aβ are the causative agents for the infectivity enhancing effect. To achieve this, synthetic human Aβ(1–42) peptide (purity > 95%) was purchased from Bachem (Bubendorf, Switzerland). Lyophilizated Aβ(1–42) was dissolved to 1 mM with hexafluoroisopropanol (HFIP) overnight at room temperature (RT). Prior to use, HFIP was evaporated using a SpeedVac Concentrator 5301 (Eppendorf; Hamburg, Germany) at RT. For preparation of Aβ(1–42) fibrils, the Aβ pellet was dissolved in PBS (phosphate buffered saline: 140 mM NaCl, 2.7 mM KCl, 10 mM Na_2_HPO_4_, and 1.8 mM KH_2_PO_4_, pH 7.4) to 1 mM and incubated four days at 37°C without shaking. To remove all soluble Aβ, the samples were washed by centrifugation and redissolved in PBS. For preparation of Aβ(1–42) mono- and oligomers, the Aβ pellet was dissolved in SEC buffer (size exclusion chromatography buffer: 50 mM NaPi pH 7.4, 150 mM NaCl) and purified using size exclusion chromatography (Figure 1D). To test the different Aβ conformers for their infectivity enhancement potential, we used TZM-bl reporter cells that harbor a luciferase and a β-galactosidase expression cassette under the control of the HIV-1 LTR promoter, which are activated in infected cells due to expression of the HIV-1 trans-activator of transcription (Tat). These reporter cells were infected with equal amounts of the dual-tropic (R4 and R5) HIV-1 PI 952 [11] either in presence or absence of Aβ(1–42) monomers, oligomers or fibrils. For luciferase measurements, cells were rinsed in PBS and dispensed in passive lysis buffer (PLB) and shaken for 15 min at RT. Luciferase activity of cell lysates was measured by adding Beetle-Juice (p.j.k; Kleinblittersdorf, Germany) using an Infinite 200 PRO multimode reader (Tecan; Männedorf, Switzerland). We observed that Aβ(1–42) fibrils (Figure 1A and B) but not mono- or oligomers (Figure 1E and F) were able to enhance HIV-1 infection of TZM-bl cells. The enhancing effect of Aβ(1–42) fibrils on HIV-1 infectivity was observed at a concentration of 2 μg/ml and augments with increasing Aβ(1–42) fibril concentrations, whereas Aβ(1–42) fibrils alone had no effect on luciferase expression of TZM-bl cells (Figure 1C). In agreement with Münch et al. [3], but in contrast to Wojtowicz et al. [2], we did not observe any enhancing effect on HIV-1 infection when using Aβ(1–40) fibrils (Innovagen; Lund, Sweden) irrespective of whether these were incubated for four or six days of oligomerization under the same conditions as described above (Figure 2). The reason for this discrepancy was already discussed by Münch et al. arguing that amyloid fibrils composed of the same protein can show different conformations with distinct phenotypes [12].

**Figure 3:**
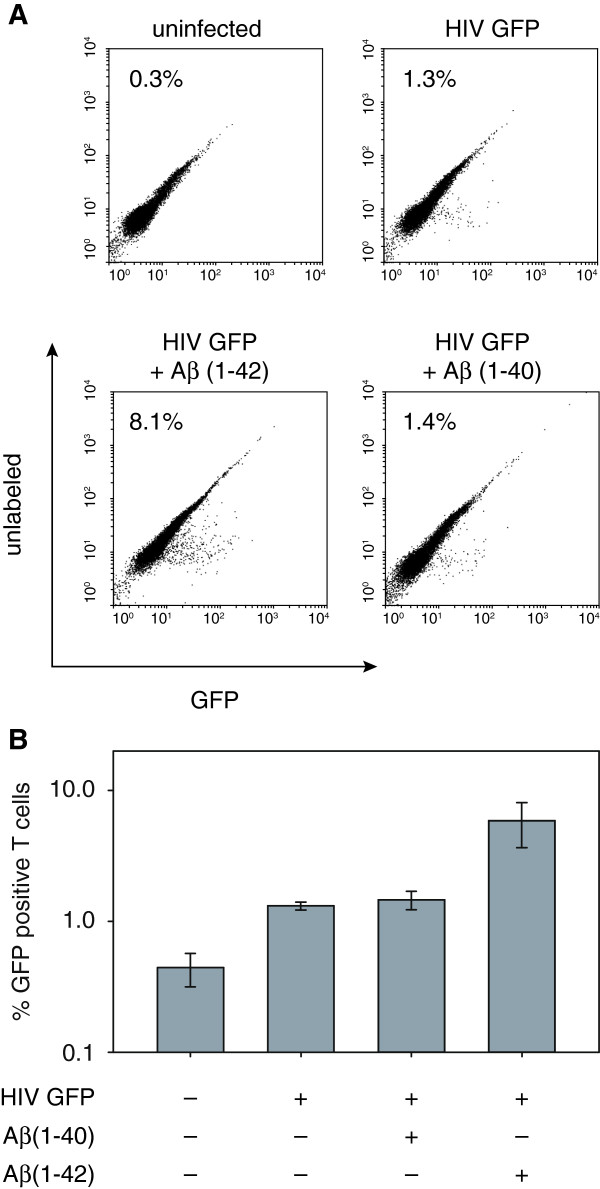
**Aβ(1–42) peptide boosted infectivity of Molt-4 T cells. (A)** Equal amounts of the R4 tropic HIV-1 NL4-3 lab strain expressing a NEF-GFP fusion protein were pre-incubated for 5 min at RT with PBS only, Aβ(1–40) or Aβ(1–42) fibrils [10 μg/ml]. Subsequently, Molt-4 T cells were infected with the pre-treated viruses and the percentage of HIV-1 infected (GFP positive) cells was assayed by FACS analysis 48 h post infection. **(B)** The percentage of HIV-1 infected (GFP positive) cells.

To analyze whether the infectivity boosting effect of Aβ(1–42) but not Aβ(1–40) fibrils was cell type specific, we applied our approach also to the HIV-1 susceptible Molt-4 T cells [13,14]. Equal amounts of an R4 tropic HIV-1 NL4-3 derivate, which expresses a NEF-GFP fusion protein, were pre-incubated for 5 min at RT with Aβ(1–42) or Aβ(1–40) fibrils (10 μg/ml) and PBS as a control, respectively. Subsequently, the pre-treated viruses were used to infect Molt-4 T cells and the percentage of infected (GFP positive) cells was assayed by FACS analysis by using FACSCalibur (BD; Franklin Lakes, USA) 48 h post infection. As expected, treatment with Aβ(1–42) but not with Aβ(1–40) fibrils resulted in ~ six-fold higher percentage of GFP positive T cells when compared to PBS treated cells indicating that Aβ(1–42) specifically enhances viral infectivity also in T cells (Figure 3).

**Figure 4:**
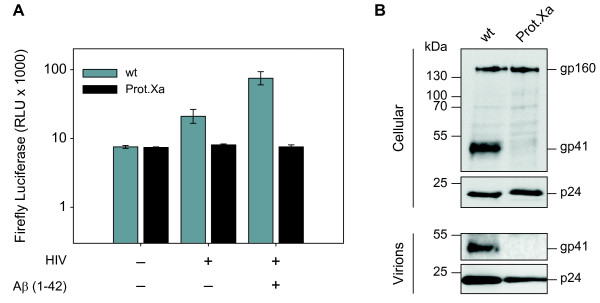
**The fibril-mediated enhancement of HIV-1 infectivity is dependent on the membrane fusion activity of gp41. (A)** TZM-bl cells were infected with R4 tropic HIV-1 strain NL4-3 (wt) or protease cleavage site mutant derivate (Prot.Xa), which were pre-incubated for 5 min at RT with Aβ(1–42) fibrils (50 μg/ml). Infection-induced luciferase activity was assayed 48 h post infection. **(B)** Immunoblot analysis of HEK 293T cells transfected with pNL4-3 or the protease cleavage site mutant pNL Prot.Xa by using Chessie 8 antibody (NIH) for gp160/gp41 analysis [15] and the p24 specific antibody (D7320, Aalto Bioreagents Ltd. Dublin, Ireland) for capsid protein detection. Virions were pelleted by using sucrose centrifugation as described before [16].

We further addressed the question of whether the boosted viral infectivity was also dependent on the membrane fusion activity of the gp41 N-terminus. Therefore, we transfected HEK 293T cells with pNL4-3 or the protease cleavage site mutant pNL Prot.Xa that prevents the Env glycoprotein mediated membrane fusion (kindly provided by Valerie Bosch) and performed immunoblot analysis of cellular as well as virion associated gp160/gp41 by using Chessie 8 antibody [15]. Virions were pelleted by using sucrose centrifugation as described before [16]. Next, we incubated TZM-bl cells with wildtype and mutant virus. By adding Aβ(1–42) fibrils, the defect in viral entry could not be restored indicating that the fibril-mediated enhancement was also dependent on the membrane fusion activity of gp41 (Figure 4).

**Figure 5:**
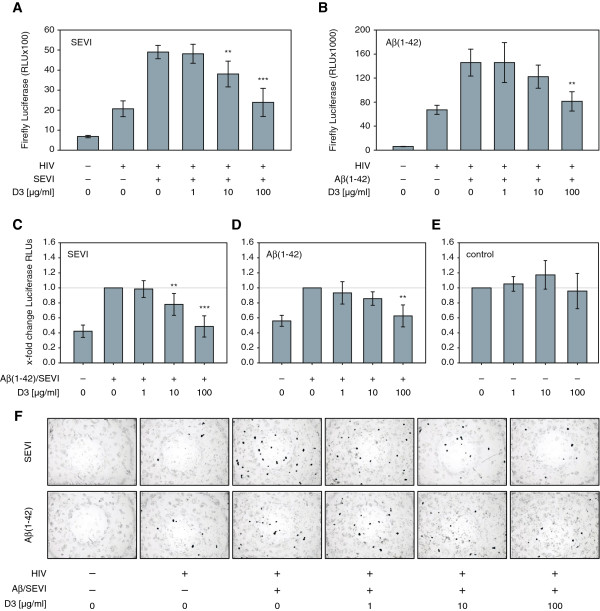
**Amyloidogenic inhibitor D3 reduces peptide boosted infectivity. (A and B)** TZM-bl reporter cells were infected with equal amounts of the dual-tropic HIV-1 lab strain NL4-3 PI 952 [11] in the presence of 10 μg/ml SEVI **(A)** or Aβ(1–42) fibrils **(B)**. As indicated, the fibrils were preincubated with D3 (0, 1, 10 or 100 μg/ml) prior to infection. Luciferase activity of infected cells was assayed 48 h post infection. **(C and D)** X-fold change Luciferase RLUs from **(A and B)**. **(E)** X-fold change Luciferase RLUs of infected TZM-bl cells, which were infected with viruses that were treated with D3 in the absence of fibrils at the indicated concentrations. **(F)** X-Gal staining of TZM-bl reporter cells that were infected as described above **(A and B)**. (*** p < 0.001, ** p < 0.01 referred to fibril treated and infected cells).

We next examined whether the peptide boosted enhancement can be reduced by pre-treatment with the non-cytotoxic Aβ fibril inhibitor D3 [17] (JPT; Berlin, Germany), which is a D-enantiomeric peptide (RPRTRLHTHRNR). SEVI and Aβ(1–42) fibrils (10 μg/ml) were pre-treated with D3 and the mixture was used to boost the infection of TZM-bl cells as described above. Following an incubation time of 48 h, the infectivity was determined by luciferase measurement and X-Gal staining (Figure 5). While SEVI and Aβ(1–42) fibrils were able to boost viral infection at similar amounts, already equimolar doses of D3 (10 μg/ml) were sufficient to significantly reduce the enhancing effect of SEVI (Figure 5A and 5C). By adding higher amounts of D3 (100 μg/ml), luciferase expression was further reduced to levels comparable with PBS treated control samples (Figure 5A, 5C and 5F). Similarly, the Aβ(1–42) boosted infection could be reduced. By adding ten-fold higher concentration of the inhibitor D3 (100 μg/ml), the infection rate of Aβ(1–42) boosted virions was significantly reduced to levels of PBS treated viruses (Figure 5B, 5D and 5F). To further control whether the reducing effect of D3 on fibril boosted infectivity was indeed due to the fibril-D3 interaction, we also pre-incubated virus containing supernatants with D3 in the absence of fibrils and then infected TZM-bl cells. As shown in Figure 5E, when infected in the absence of fibrils the cellular luciferase activity was not affected.

HIV-1 entry in female mucosa is restricted and requires overcoming at least three hurdles. These are to breach the mucosal barrier and get through the epithelium, infection and replication in sub-epithelial mononuclear cells and the initiation of a systemic infection in the lymph nodes [18]. Since genital mononuclear cells, including dendritic cells (DCs), macrophages and lymphocytes are susceptible to HIV-1 *in vivo*[18], amyloid fibrils might help HIV-1 to penetrate the mucosa and to reach these cells. Thus, treatment with D3 could inhibit the first sub-epithelial contact and prevent viral spreading.

In addition to its activity to enhance the infectivity of HIV-1 in semen, amyloids could play an important role in the progression of AIDS dementia complex (ADC) also known as HIV encephalopathy, which develops in between 20% and 30% of HIV patients in the course of infection. Interestingly, the formation of Aβ aggregates and fibrils is thought to precede the clinical symptoms of AD by three to four decades, and such fibrils may therefore be present in many mid-aged people. Since, the D-amino acid peptide D3 drastically reduces plaque load [17] and cognitive deficits even in orally D3 treated AD transgenic mice [10], it might be suitable to additionally reduce the fibril boosted HIV-1 infectivity *in vivo*.

In conclusion, the application of D3 may reduce SEVI-induced enhancement of viral infectivity of HIV-1 and the vulnerability of the central nervous system of HIV infected individuals. Thus, D3 seems to be suitable as therapeutic and prophylactic drug expanding the current HIV-intervening repertoire of antiretroviral compounds.

## Abbreviations

Aβ: Amyloid-beta; CA: Capsid; ELISA: Enzyme-linked immunosorbent assay; FACS: Fluorescence activated cell sorting; GFP: Green fluorescent protein; HIV-1: Human immunodeficiency virus type 1; PBS: Phosphate buffered saline; PLB: Passive lysis buffer; RLU: Relative light units; SEC: Size exclusion chromatography; SEVI: Semen-derived enhancer of virus infection.

## Competing interests

The authors declare that we have applied for a patent related to the content of this manuscript.

## Authors’ contributions

MW conceived, designed, and performed HIV-related infection and readout experiments, performed the statistical analysis and drafted the manuscript. AK carried out fibril preperations. YC carried out fibril preperations. AF conceived the study, and participated in its design and coordination and helped to draft the manuscript. DW conceived the study, and participated in its design and coordination and helped to draft the manuscript. HS conceived the study, and participated in its design and coordination and helped to draft the manuscript. All authors read and approved the final manuscript.
